# In vivo comparison of braided (Accero) and laser-cut intracranial stents (Acclino, Credo): evaluation of vessel responses at subacute and mid-term follow-up in a rabbit model

**DOI:** 10.1007/s10856-020-06460-z

**Published:** 2020-12-03

**Authors:** Ruben Mühl-Benninghaus, Toshiki Tomori, Stefanie Krajewski, Philipp Dietrich, Andreas Simgen, Umut Yilmaz, Christoph Brochhausen, Mara Kießling, Wolfgang Reith, Giorgio Cattaneo

**Affiliations:** 1 Department of Neuroradiology, Saarland University Hospital, Homburg/Saar, Germany; 2 Department of Thoracic, Cardiac and Vascular Surgery, University Hospital, Tuebingen, Germany; 3 Department of Pathology, University of Regensburg, Regensburg, Germany; 4 Institute for Biomedical Engineering, University of Stuttgart, Stuttgart, Germany

**Keywords:** In vivo comparison of braided and laser-cut stents, Thromboembolism, Neointima

## Abstract

This study aimed to investigate in vivo two stent technologies, with particular emphasis on thrombogenicity and inflammatory vessel remodeling processes. The micro-stents tested in this study were developed for intracranial aneurysm treatment. In our study twelve, New Zealand white rabbits were divided into two groups: 18 laser-cut stents (LCS) and 18 braided stents (BS) were impanated without admiration of antiplatelet medication. Three stents were implanted into each animal in the common carotid artery, subclavian artery, and abdominal aorta. Digital subtraction angiography was performed before and after stent implantation and at follow-up for the visualization of occurring In-stent thromboembolism or stenosis. The Stents were explanted for histopathological examination at two different timepoints, after 3 and 28 days. Angiographically neither in-stent thrombosis nor stenosis for both groups was seen. There was a progressive increase in the vessel diameter, which was more pronounced for BS than for LCS. We detected a higher number of thrombi adherent to the foreign material on day 3 for BS. On day 3, the neointima was absent, whereas the complete formation observed was on day 28. There was no significant difference between both groups regarding the thickness of the neointima. The in vivo model of our study enabled the evaluation of blood and vessel reactions for two different stent technologies. Differences in vessel dimension and tissue around the stents were observed on day 28. Histological analysis on day 3 enabled the assessment of thrombotic reactions, representing an important complementary result in long-term studies.

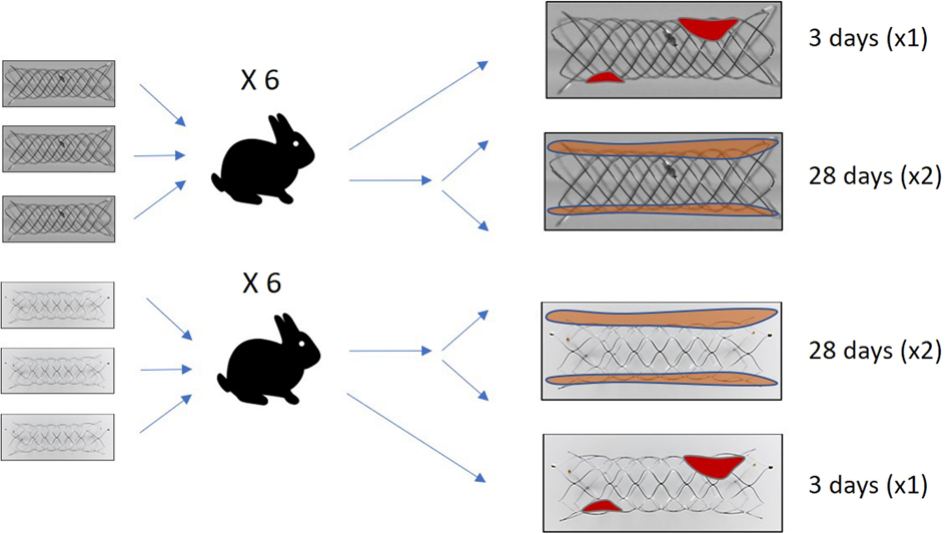

## Introduction

Stent-assisted coiling of wide-necked intracranial aneurysms aims to prevent coil prolapse into the parent vessel, enhancing vessel remodelling processes. Although this neurovascular technique has proven effective and safe in several studies, the presence of foreign material within the vessel lumen is associated with clinical complications. Namely, thromboembolic events and delayed stenosis have been reported and represent an important factor threatening the outcome of the procedure [1–4].

A better understanding of the cellular effects of stent design on blood and vessel reactions can potentially contribute to both product design development and procedural planning [5, 6]. State-of-the-art implants can be divided into two groups with regard to manufacturing and design features: laser-cut stents (LCS) and braided stents (BS). LCS involves removing material from a tube, creating openings surrounded by struts that deform from the radially compressed configuration to the expanded configuration. In contrast, BS comprises multiple wires interwoven into a cylindrical shape. The design of BS reduces deformation of the wire by allowing relative sliding between wires. The divergent manufacturing processes result in consistent differences between both stent technologies, particularly regarding stent elements (squared struts in LCS vs. round wires in BS), stent profile (thicker in BS because of crossing wires at interconnecting points), and radial expansion force (high in both stents, depending on construction parameters and size) Moreover, surface finishing is usually different in the two technologies [7].

The present study evaluated LCS and BS in a novel in vivo animal model, facilitating histopathological examination at subacute and mid-term follow-up, with emphasis on vessel response and neointima formation. To the best of our knowledge, this study represents the first in vivo comparison of two state-of-the-art technologies for intracranial stents and a novel analysis of harvested stented vessels at a subacute phase.

## Materials and methods

### Implants

Both the LCS (Acclinoflex®/Credo®) and BS (Accero®) used in this study are clinically available (Acandis GmbH Pforzheim, Germany) and compare the superelastic nickel titanium alloy Nitinol^©^. LCS have six openings around the circumference and a wall thickness of 50 µm. The struts have an alternating width of 40 and 32 µm, resulting in an asymmetrical structure for improved flexibility. Three radiopaque markers are placed at each end. For this study, LCS with a diameter of 4.5 mm and lengths of 15 mm or 20 mm were used.

The BS used have a diameter of 2.5 mm or 4.5 mm. The stent comprised a 50 µm single wire bent at each end in multiple loops, resulting in six and nine openings around the circumference for both sizes, respectively. Implant thickness at the wire crossing points was 100 µm. The open wire ends were connected with a radiopaque marker in the middle of the stent.

### Animal studies

The study was approved by the University’s animal welfare committee and conducted in accordance with the institutional animal experiment guidelines. Twelve rabbits were implanted with 36 stents, either of LCS (*n* = 18) or BS (*n* = 18) type. Because of design-specific features discernible during the procedure, both groups were not blinded. Antiplatelet therapy was not provided before, during, or after stenting. The right femoral artery was surgically exposed under general anaesthesia and a 4 F sheath inserted. A dose of 300 U heparin was administered intravenously during the procedure. Stents were implanted over the wire. Opening of the markers confirmed adequate positioning and expansion.

**Fig. 1:**
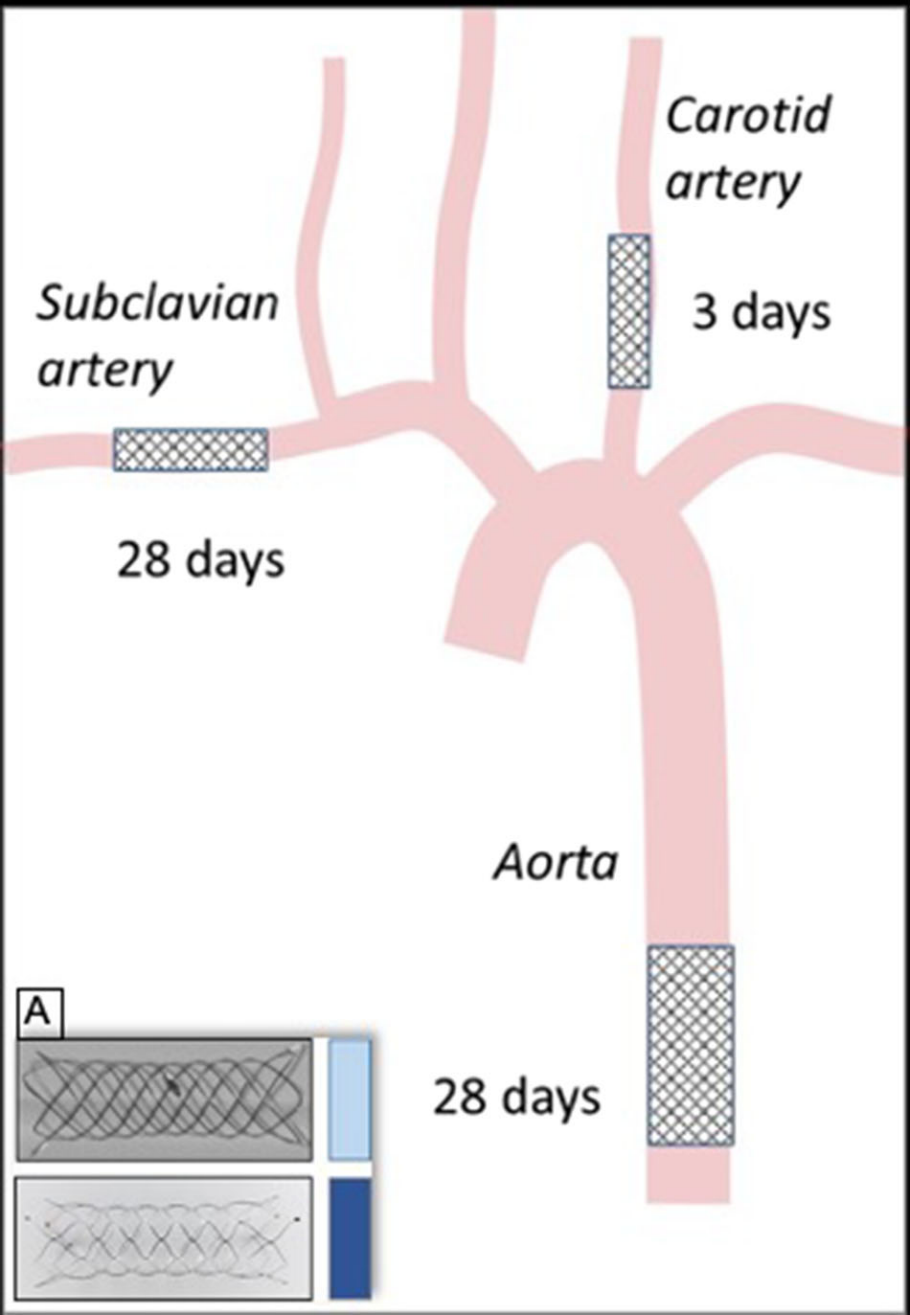
Schematic drawing of the positioning of the three stents in the common carotid artery, the subclavian artery, and the abdominal aorta. **a** Picture of the two analysed stents: braided Accero® (top) and laser-cut Acclinoflex®/Credo® (bottom)

Target vessels for stent placement are depicted in Fig. 1. Two stents were placed in the right subclavian artery (SA) distal to the orifice of the vertebral artery and in the left common carotid artery (CCA), respectively. These vessels were selected due to the small lumen diameter of ~2 mm, representing a worst-case alignment with regard to stent thickness/vessel lumen ratio. To provide an identical number of cells along the circumference, and, therefore, a similar metal coverage ratio, different stent sizes were implanted. For BS, 2.5 × 10 mm was used, which was intended for a vessel diameter of 1.5–2.5 mm, whereas 4.5 ×15 mm was used for LCS, which was intended for a vessel diameter of 2.5–4 mm. The stent length for BS was 12 mm and for LS 17.7 mm in vessels with a diameter of 2 mm. The stent length increased in BS for smaller vessel lumens (internal data), reducing the length difference between the two stents. A third stent was implanted in the abdominal aorta (AA) with a stent diameter of 4.5 mm for each group and a length of 20 and 15 mm for LCS and BS, respectively. The stent length for LCS was 19.7 mm and for BS 17.7 mm in vessels with a diameter of 3.5 mm.

Angiographic control was performed using a 4 F diagnostic catheter after implantation. After catheter retrieval, the femoral artery was ligated, and the animals allowed to recover.

**Fig. 2:**
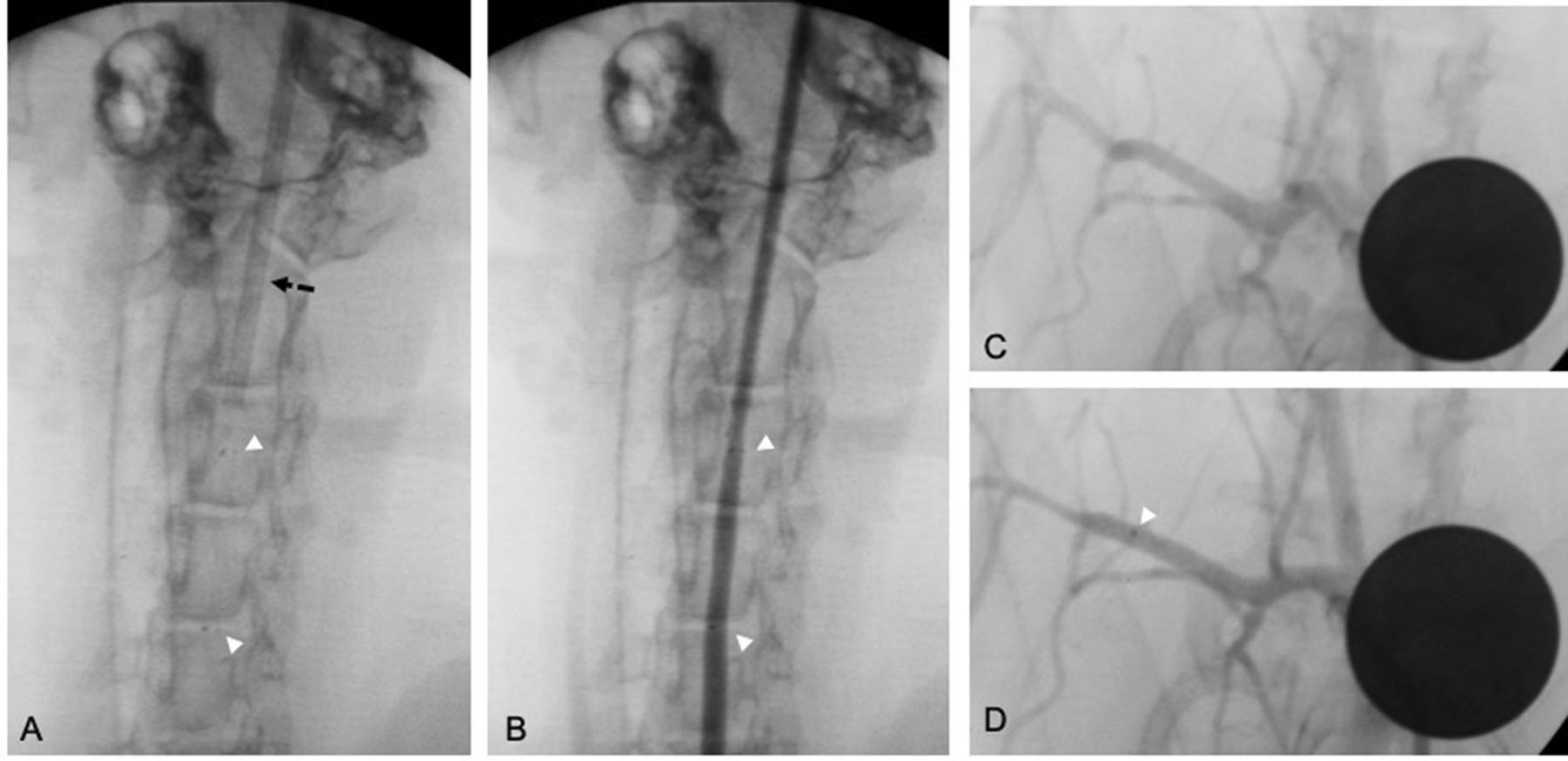
Unsubtracted angiograms of the left common carotid artery (CCA) (**a**, **b**) and subclavian artery (SA) (**c**, **d**) implanted in a retrograde fashion through a sheath (dashed arrow). White arrowheads highlight stent markers

Follow-up controls were conducted at 3 days (subacute) and 28 days (mid-term) to assess blood and vessel responses (Fig. 2).

#### Subacute follow-up at day 3

After surgical exposure of the left carotid artery, a 4 F sheath was placed distal to the stent for retrograde digital subtraction angiography (DSA) of all three vessels. A retrograde approach was performed due to the limited arterial accesses. Following angiography, the left CCA with the stent was resected for histological analysis. The artery was ligated proximally, and the animals allowed to recover.

#### Mid-term follow-up at day 28

DSA of the two remaining stents was performed using a 4 F sheath placed in the right femoral artery, as described above for day 3. Animals were subsequently euthanized via lethal injection of pentobarbital and vessels removed for histology.

For stents inserted in the SA and the AA, diameter measurement was performed directly before and after stent implantation, on day 3, and on day 28. Removed specimens were fixed in formalin for histological analysis.

## Data analyses

### Angiographic evaluation

Vessel diameters in the middle section of the stents obtained from the DSA were evaluated by three experienced interventional neuroradiologists (RMB, UY, AS) on consensus in posterior-anterior projection using the post-processing software OsiriX (Pixmeo, Switzerland). The vessel diameter was averaged for each vessel, stent type, and follow-up time, including before implantation, on day 3, and on day 28 s (the latter only for the SA and the AA). Patency of the branch arteries covered by the devices was also assessed at follow-up.

### Histopathology

Histopathological processing and histomorphometry are described in the Supplementary material. Analysis of the subclavian arteries and aorta included leukocyte and giant cell counts, dehiscence, media layer compression, and in-stent thrombosis. Due to the earlier explantation time, CCA analysis focused on fresh and residual thrombi in the vessel wall, the presence of thrombotic material surrounding stent struts, and in-stent thrombosis.

### Statistical analyses

Statistical analysis was performed using GraphPad Prism (version 6.01, GraphPad Software, U.S.A.). Data for the two groups were analysed with an unpaired Student’s *t* test. One-way ANOVA with Holm-Sidak´s post hoc correction was used for comparisons of more than two groups. Continuous variables are expressed as means and standard deviations. A paired *t* test was used to determine the significance of any difference in the changes within each group. A two-sided *P* ≤ 0.05 was statistically significant.

## Results

### Angiographic evaluation

Stent delivery and deployment was successful in all twelve rabbits. No cases of stent migration were observed. There was no stenosis or in-stent thrombosis during follow-up for both stent types. Branch artery occlusion was not observed.

Average vessel diameter before stent implantation and at follow-up times as well as the percentage increase relative to pre-implantation time are shown in Table 1.

**Table 1:** Angiographic measurement of vessel diameter and progressive vessel lumen increase

Follow-up	Carotid artery	Subclavian artery	Abdominal aorta
Laser cut stent (LCS)			
Vessel lumen diameter (mm)			
Before impl.	1.92 ± 0.19	1.92 ± 0.21	3.50 ± 0.33
Day 3	2.03 ± 0.23	2.00 ± 0.22	3.52 ± 0.34
Day 28	–	2.00 ± 0.22	3.55 ± 0.26
Vessel lumen dimeter increase (%)			
Day 3	6.0%	4.4%	0.5%
Day 28		4.4%	1.6%
Braided stent (BS)			
Vessel lumen diameter (mm)			
Before impl.	1.88 ± 0.23	1.97 ± 0.16	3.53 ± 0.37
Day 3	2.08 ± 0.12	2.12 ± 0.15	3.57 ± 0.35
Day 28		2.20 ± 0.11	3.60 ± 0.32
Vessel lumen diameter increase (%)			
Day 3	11.6%	7.8%	1.0%
Day 28		12.4%	2.1%

**Fig. 3:**
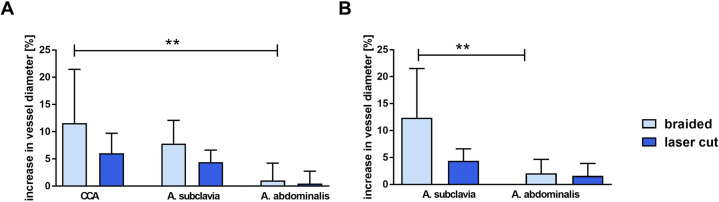
Percentage of increase in vessel diameter at 3 days (**a**) and 28 days (**b**) of follow-up. In the case of BS implantation, there was a significantly higher diameter increase in the common carotid artery (CCA) compared to the abdominal aorta (AA) after 3 days (**P = 0.006) as well as in the subclavian artery (SA) compared to the abdominal aorta (AA) after 28 days (**P = 0.0039)

There was a trend toward a progressive increase of vessel diameter in all arteries during follow-up, except for the SA with the LCS between days 3 and 28. BS were observed to generally result in greater vessel lumen increase over the follow-up period for all arteries compared to LCS (Table 1). The maximum vessel diameter increase following BS insertion was observed in the SA at 28 days follow-up in contrast to the CCA at 3 days follow-up. Furthermore, vessel lumen increase was significantly higher in the CCA and SA compared to the AA at 3 and 28 days follow-up, respectively (*P* = 0.006 and *P* = 0.0039) (Table 1 and Fig. 3). For LCS, a maximum vessel diameter increase of 6% was observed after implantation in the CCA at 3 days follow-up.

### Histomorphology

**Fig. 4:**
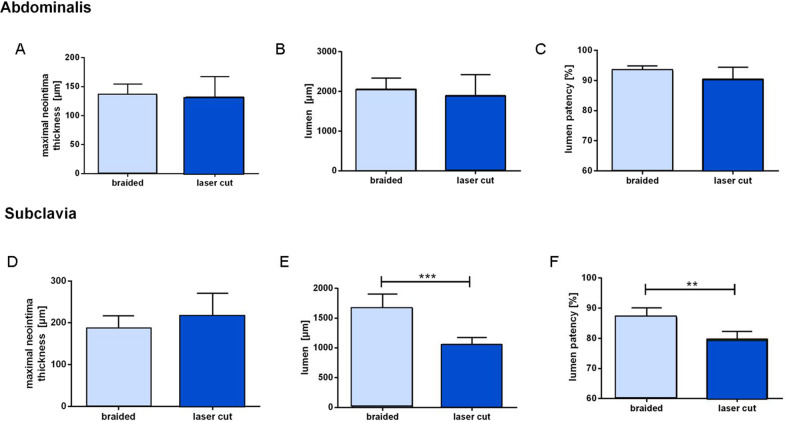
Histomorphological analysis of neointimal thickness (**a**, **d**), vessel lumen (**b**, **e**), and lumen patency ratio (**c**, **f**) for the abdominal aorta (AA) and subclavian artery (SA) at 28 days follow-up, showing a significantly higher lumen diameter (***P = 0.0002) and patency (**P = 0.0014) for braided stents in the SA

In both the AA and SA, there was no significant difference in the maximum neointimal thickness between the two groups (*P* = 0.085, *P* = 0.027) (Fig. 4). Comparison of the BS and LCS types only revealed a significantly higher vessel lumen diameter (*P* = 0.0002) and lumen patency (*P* = 0.0014) in the SA.

### Histopathology at day 3 (CCA)

**Fig. 5:**
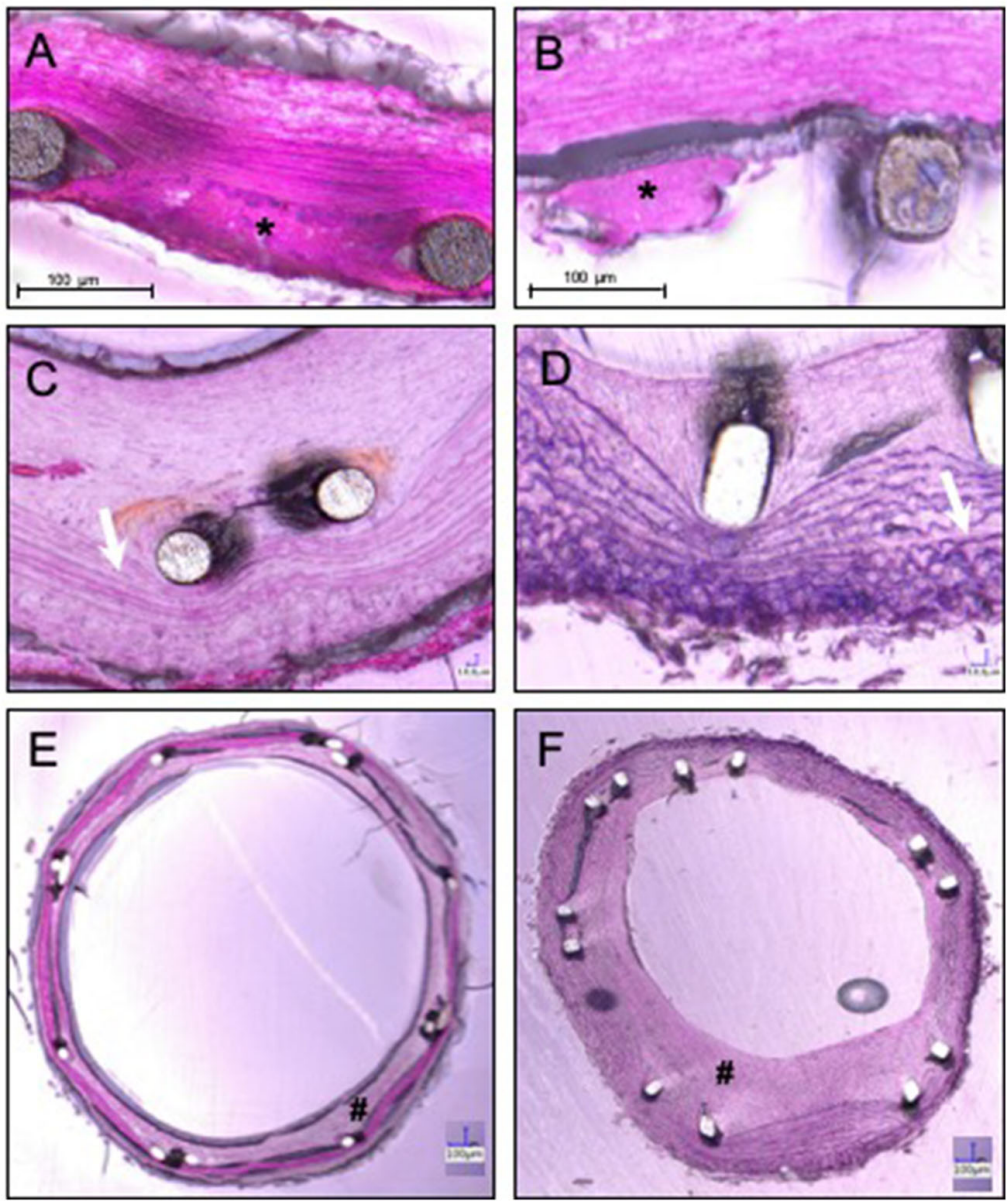
Representative histological images of a transverse section of a laser-cut stent (LCS; **a**) and a braided stent (BS; **b**) in the subclavian artery at 3 days and 28 days follow-up (**c**, **d**). An asterisk shows thrombus formation. White arrows show compression of elastic fibres in the tunica media. Representative histological images of a transverse section of an LCS (**e**) and a BS (**f**) in the subclavian artery at 28 days follow-up showing intimal hyperplasia. **#** shows the neointima

At day 3, thrombus material was found in the strut regions of the explanted devices (Table 2 and Fig. 5), with a considerably higher frequency in BS compared to LCS. Adherent clot material was observed in 100% of the BS structs compared to 58% of LCS struts. Residual thrombi were found in three BS within the clot and were in direct contact with the strut surface. A thrombus in the lumen was present in one stent in the LCS group, with focal vessel wall contact containing an extensive accumulation of fresh erythrocyte. A residual thrombus was not observed within the vessel wall.

**Table 2:** Histopathology of the left carotid artery at day 3

	Left carotid artery (day 3)	
	LCS	BS
Struts with clot material	58%	100%
Fresh thrombus	0	0
Residual thrombus	0	3 (within clot)
Thrombus within the lumen	1	0

### Histopathology at day 28 (AA and SA)

Dehiscence was frequently observed and usually commenced in the stent strut region, developing at the interface between the neointima and the media layer, and, in some cases, into the media to the media-adventitia border. In the AA, dehiscences (both focal and widespread) were observed in only one vessel with an LCS compared to 5 of 6 vessels treated with BS. In the SA, dehiscence was present in all vessels and was widespread in three LCS and five BS.

Inflammation and foreign body reactions were rare (Table 3), with only one lymphocyte found in a BS implanted in the AA and one giant cell in an LCS inserted in the SA. In both groups, media compression was present in all samples.

**Table 3:** Histopathology at day 28 of the abdominal aorta and subclavian artery

	Abdominal aorta		Subclavian artery	
	LCS	BS	LCS	BS
Inflammatory cells	0	1	0	0
Giant cells	0	0	1	0
Media compression	all	all	all	all
Dehiscence (only focal)	0	2	3	1
Dehiscence (widespread)	1	3	3	5

## Discussion

The present study reports the first systematic in vivo comparison of two main neurovascular stent systems, LCS and BS. Despite the expanding use of flow diverters, which reconstruct the lumen through flow modulations, stents can still provide coil support in wide-necked aneurysms, with a considerably lower metal coverage than that in flow diverters, therefore potentially resulting in fewer thromboembolic complications.

Various clinical studies have investigated both BS and LCS [8–11]. The risk of thromboembolic complications related to both stent types ranges between 4 and 8% [12–14]. However, there is very little data available comparing the two technologies within the framework of an experimentally [15, 16]. Concurrently, studies on in vivo animal models of neurovascular stents are rare. Krings et al. [17] compared different approaches to treat cerebral aneurysms in created aneurysms in 30 rabbits using porous LCS, covered stents (polyurethane shore 60 D), and stent (porous LCS) assisted coiling. At a follow-up time of 3 and 6 months. They observed a higher aneurysm occlusion rate in the covered stent group compared to both groups treated with porous stents, with and without coils. Moreover, while covered stents presented a mild neointima thickening and one parent vessel occlusion (10%), in-stent-stenosis was more frequent in porous stents, amounting up to 40%. Another study group investigated the alteration of blood flow dynamics after FD and laser-cut stent implantation in the rabbit aneurysm model. They observed prolonged blood stagnation in the aneurysm sac in the FD group [18]. However, the appearance of thrombus formation or inflammatory reactions was not assessed when comparing the different stent types in vivo. In both studies, follow up times between 1 and 6 months addressed middle term vessel reaction, while acute vessel injury and thrombosis were not mattered of investigation.

The present study focused on biocompatibility with regard to blood and vessel responses, comparing the two stent technologies in a rabbit model. To achieve this, a worst-case animal model was used, with stent implantation in small arteries without antiplatelet therapy. This scenario represents a critical condition, potentially provoking further thromboembolic and inflammatory reactions to facilitate a comparison of the two stent groups.

Explanting of the CCA after 3 days and both the AA and SA after 28 days enabled two follow-up periods in the same animal, reducing the number of animals sacrificed. Both follow-up times were selected with the expectation of observing acute reactions at day 3 post-implantation as well as neointimal thickness at day 28. Interestingly, neointimal thickness was well developed and did not increase considerably beyond the highest level observed after one month, as previously reported [19, 20].

There was no concern about angiographic parent vessel and branch artery patency. In-stent thrombosis and stenosis were not observed at angiographic follow-up. Moreover, vessel diameter measurements showed enlargement in all vessels at almost all times, which was not significant. In comparison to pre-implantation values, this enlargement was considerably higher for BS compared to LCS, exceeding 10% in the CCA at 3 days (12.4%) and in the SA at 28 days (11.6%). Considering the reciprocal and disproportionate relationship between vessel resistance and the diameter, the maximum measured enlargement of 12.4% would lead to a decrease in blood flow resistance to ~60%. However, it remains unknown whether this decrease in resistance has the potential to impact blood flow in vessels in clinical conditions, since the flow rate results from numerous serial vascular resistances, with the stented vessel region representing a short part en route to the brain. Although the study did not include a control group without stent implantation, the considerable vessel enlargement observed on day 3 implied that this effect was growth-dependent.

Histomorphology showed a significantly greater lumen diameter in the SA treated with a BS compared to LCS, confirming angiographic measurements. Interestingly, the patency ratio was also significantly higher in BS. However, this observation was attributable primarily to the greater vessel enlargement and not to neointimal formation, which was comparable in both groups. Furthermore, the absolute vessel diameter values measured by histomorphological evaluation were considerably smaller than those measured in angiography in both the SA and the AA. This discrepancy could be based on vessel behaviour during the explanation phase, potentially attributable to shrinkage due to intraluminal pressure reduction or spastic reaction.

After 28 days, considerable inflammation and foreign material reactions in stented vessels were not observed histopathologically. Correspondingly, only two vessels demonstrated a mild reaction. The media layer was compressed in all samples, with no clear differentiation between the two groups. The most pronounced effect observed at the longer follow-up period was the presence of dehiscence, which emerged in the strut region. This effect appears most pronounced in BS in both the SA and the AA. The reason for this finding remains open to debate; however, the interplay of wire movement could cause a paucity of cells in place of tissue in the region close to the wires. Furthermore, different expanding force and frictional actions of the structure relative to the vessel wall could affect cell proliferation and extracellular matrix formation. Since both stunt devices have a similar electropolished surface, we do not believe there is a biochemical reaction at the surface that is attributable to this phenomenon.

One vessel was explanted per animal 3 days after stent implantation. This was performed because an acute tissue response can also be potentially responsible for further priming the thromboinflammatory cascade, and, therefore, tissue appearance [21, 22]. The presence of thrombi at the stent differed between the LCS and BS types. All stent struts presented an adherent thrombus in the BS group compared to the LCS group (58%). Moreover, a residual thrombus at the interface between the material and the thrombus was observed in three specimens in BS, suggesting an endothelial cell injury as a potential cause for thrombi formation. Mechanisms of thrombus formation have been proposed to include fluid dynamic disturbance with blood stagnation as well as high shear stress areas [23], which could be pronounced in BS because of their higher profile at the intersection between two wires. Finally, the combination of tissue damage, blood stagnation, and blood-material contact can lead to acute thrombosis.

This study has several limitations. One primary limitation concerns the size of the stunts, which differed in the SA for both stents. A smaller BS with a diameter of 2.5 mm was selected because of the identical number of cells (6) along the stunt circumference than an LCS with a diameter of 4.5 mm. Stent dimensions affect foreshortening as well as radial force on the vessel wall. Regardless of the dimensions, an identical mechanical behaviour cannot be ensured, particularly because of the higher axial compressibility of a BS. Therefore, stents were implanted with similar geometrical characteristics. Another limitation is the lack of randomization and non-blinded nature of the study. Furthermore, only the middle section of each stent was considered for histopathological analysis, neglecting potential effects at the stent ends. Finally, the constitution and surrounding tissue of rabbit extracranial vessels differ from that of human intracranial vessels. In particular, the SA is highly exposed to leg movement in the rabbit, presumably producing more mechanical solicitations than vessels in the intracranial space.

## Conclusion

A systematic comparison of two neurovascular stent technologies was performed in a new animal model in the absence of antiplatelet therapy, including two histological follow-up times (at days 3 and 28). No stenosis or in-stent thrombosis was detected during follow-up for both stent types. Angiography revealed a more marked vessel size enlargement in the BS compared to the LCS at both follow-ups, which was confirmed by histomorphological analysis at 28 days. Thrombus formation was pronounced on BS wires than on LCS struts on day 3, which failed to result in thrombosis or increased neointimal thickness on day 28. The results support the understanding of device and vessel responses and demonstrated the feasibility of a model that facilitates further investigations into neurovascular technologies in the future.

## Supplementary information

Supplementary Material

## Abbreviations

AA: abdominal aorta
BS: braided stent
CCA: common carotid artery
DSA: digital subtraction angiography
LCS: laser-cut stent
SA: subclavian artery
