# Correction to: In vivo comparison of braided (Accero) and laser-cut intracranial stents (Acclino, Credo): evaluation of vessel responses at subacute and mid-term follow-up in a rabbit model

**DOI:** 10.1007/s10856-021-06518-6

**Published:** 2021-04-11

**Authors:** Ruben Mühl-Benninghaus, Toshiki Tomori, Stefanie Krajewski, Philipp Dietrich, Andreas Simgen, Umut Yilmaz, Christoph Brochhausen, Mara Kießling, Wolfgang Reith, Giorgio Cattaneo

**Affiliations:** 1 Department of Neuroradiology, Saarland University Hospital, Homburg/Saar, Germany; 2 Department of Thoracic, Cardiac and Vascular Surgery, University Hospital, Tuebingen, Germany; 3 Department of Pathology, University of Regensburg, Regensburg, Germany; 4 Institute for Biomedical Engineering, University of Stuttgart, Stuttgart, Germany

Correction to: Journal of Materials Science: Materials in Medicine (2020) **31**:131

10.1007/s10856-020-06460-z published online 3 Dec 2020

The original version of this article unfortunately contained a mistake. The given names and family names of the authors were interchanged. The correct author names are Ruben Mühl-Benninghaus, Toshiki Tomori, Stefanie Krajewski, Philipp Dietrich, Andreas Simgen, Umut Yilmaz, Christoph Brochhausen, Mara Kießling, Wolfgang Reith, Giorgio Cattaneo. The original article was corrected.

