# Supplementary material for: In vivo comparison of braided (Accero) and laser-cut intracranial stents (Acclino, Credo): evaluation of vessel responses at subacute and mid-term follow-up in a rabbit model
Source: J Mater Sci Mater Med. 2020 Dec 3;31(12):131. doi: 10.1007/s10856-020-06460-z (PMC7716819; doi:10.1007/s10856-020-06460-z)
Supplement: Supplementary file 1 — Supplementary Material [file 10856_2020_6460_MOESM1_ESM.docx]

*Histopathological Processing*

The explanted devices were fixed in buffered formalin, and then dehydrated in graded alcohol. For histochemical analyses, samples were pre-infiltrated in ethanol/Technovit^®^ 7200 (1:1) and in pure Technovit^®^ 7200, both overnight at 4°C in a desiccator. For embedding, the samples were placed in a small dish, filled up with Technovit^®^ 7200, and cured with white light using an EXAKT 520 chamber (Norderstedt, Germany) for 10 hours, followed by 10 hours with UV light. Afterwards, the polymerized blocks were cut in slices with a thickness of 100-200 µm using a diamond band saw (EXAKT 300/310), followed by grinding and polishing (EXAKT 400CS). The histological slices were stained with hematoxylin and eosin (H&E). For each vessel, transversal cuts were made at three different regions (proximal, middle, and central).

*Histomorphometry*

After microscopy (VHX-500F; Keyence, Neu-Isenburg, Germany) and digitalization morphometric measurements were performed for each slice derived from the subclavian artery and aorta. Maximum and minimum neointimal thickness (NT, defined as the distance between the outer surface of each strut and the luminal border) was measured and the average value calculated. For the following measurements, the longest and shortest distances between the opposite sides of the lumen profiles were defined as axes. On each axis, the distance between the opposite sides of the lumen profiles and of the stent profile was calculated and defined as distance lumen (d_l_) and distance stent (d_s_). Lumen patency ratio was defined as (d_l_/d_s_)*100%. Lumen diameter, stent diameter, and lumen patency ratio were calculated as the average of d_l_, d_s_, and percentage stenosis ratio on both axes, respectively.
